# Pillars for prevention and control of healthcare-associated infections: an Italian expert opinion statement

**DOI:** 10.1186/s13756-022-01125-8

**Published:** 2022-06-20

**Authors:** Vincenzo Puro, Nicola Coppola, Andrea Frasca, Ivan Gentile, Francesco Luzzaro, Angela Peghetti, Gabriele Sganga

**Affiliations:** 1 Risk Management Unit, National Institute for Infectious Diseases “L. Spallanzani” - IRCCS, Rome, Italy; 2 Department of Mental Health and Public Medicine, University of Campania “Luigi Vanvitelli”, Naples, Italy; 3 Quality and Risk Management, Nomentana Hospital, Rome, Italy; 4 Department of Clinical Medicine and Surgery, Staff UNESCO Chair On Health Education and Sustainable Development, University of Naples “Federico II”, Naples, Italy; 5 Clinical Microbiology and Virology Unit, “A. Manzoni” Hospital, Lecco, Italy; 6 AOU Policlinico S. Orsola-Malpighi, Fondazione GIMBE a IRCCS - AOU, Fondazione GIMBE, Bologna, Italy; 7 Emergency Surgery and Trauma, Fondazione Policlinico Universitario A. Gemelli IRCCS, Catholic University of Sacred Heart, Rome, Italy

**Keywords:** Healthcare-associated infections, Infection prevention and control, IPC core components, Hand hygiene

## Abstract

Healthcare-associated infections (HAIs) represent a relevant problem for all healthcare facilities, because they involve both the care aspect and the economic management of the hospital. Most HAIs are preventable through effective Infection Prevention and Control (IPC) measures. Implementation and improvement of IPC programs are critical to reducing the impact of these infections and the spread of multi-resistant microorganisms. The purpose of this Expert Opinion statement was to provide a practical guide for healthcare organizations, physicians, and nursing staff on the optimal implementation of the core components of Infection Prevention and Control, as recommended by a board of specialists after in-depth discussion of the available evidence in this field. According to their independent suggestions and clinical experiences, as well as evidence-based practices and literature review, this document provides a practical bundle of organizational, structural, and professional requirements necessary to promote, through multimodal strategies, the improvement of the quality and safety of care with respect to infectious risk in order to protect the patient, facilities, and healthcare providers.

**Supplementary Information:**

The online version contains supplementary material available at 10.1186/s13756-022-01125-8.

## Introduction

Healthcare-associated infections (HAIs) are a major public health problem both because of the significant impact on patient morbidity, mortality, and quality of life and because of the significant economic burden on healthcare systems worldwide. However, most HAIs are preventable and can be reduced by up to 70% through effective Infection Prevention and Control (IPC) measures [1].

Improvements in IPC at the national and facility level are critical to the containment of antimicrobial resistance and prevention of HAIs, including outbreaks of highly transmissible diseases, through high-quality care in the context of universal health coverage [2].

In 2016, World Health Organization (WHO) published recommendations on effective IPC strategies based on systematic literature review and expert consensus summarized in eight core components, followed by a set of minimum requirements for their implementation [3–5]. According to these documents, the core components needed to improve IPC practices are: (1) IPC programme, (2) IPC guidelines, (3) IPC education and training, (4) Healthcare-associated infection surveillance, (5) Multimodal strategies, (6) Monitoring/audit of IPC practices and feedback, (7) Workload, staffing and bed occupancy and (8) Built environment, materials and equipment for IPC.

In 2017, an IPC assessment framework (IPCAF) was developed by the WHO IPC Global Unit to support the implementation of WHO guidelines on core components of IPC programs in acute healthcare facilities and to enable these facilities to self-evaluate IPC practices [6]. However, applying the 2017 IPCAF, several surveys have shown that the WHO core components are difficult to implement at the facility level [7–9].

A board of specialists agreed to draft an expert opinion paper to guide healthcare organizations, physicians, and nurses on the optimal implementation of the HAI-IPC core components, after a thorough discussion of the available evidence in this field.

The purpose of this document is to provide healthcare organisations with a practical bundle of organisational, structural and professional requirements necessary to promote, through multimodal strategies, the improvement of the quality and safety of care with regard to infectious risk, in order to protect the patient, facilities, and healthcare workers.

## Methods

Prevention and management of HAIs should focus on collaboration among all healthcare professionals with shared knowledge and widespread diffusion of best practices.

Seven different Italian professionals involved in the issue of HAIs prevention and control in their respective hospitals convened in a panel in order to identify and provide a practical set of organizational, structural, and professional requirements crucial for effective implementation of infection control programs: three infectious disease specialists, two nurses, a Director of microbiology, and a Director of emergency and trauma surgery with a well-known experience in surgical infections and sepsis.

The experts decided to draft an expert opinion paper on the prevention, surveillance, and control of HAIs in healthcare facilities, according to their independent suggestions and clinical experiences, as well as to evidence-based practices. A non-systematic review of the literature was performed and discussed by the panel.

During a first virtual meeting in May 2021, the experts outlined the HAI risk scenario and identified risk factors through a series of questions (brainstorming) to discuss the main areas of interest with the aim of producing a document as a useful support tool for all healthcare professionals working in the context of HAI prevention and monitoring processes. All feedback from the brainstorming was collected and shared via email prior to the second virtual meeting held in July 2021 where the experts wrote the outline of the paper. The experts communicated via email to prepare, discuss, and revise the document, adding boxes to the paper in which to describe some personal experiences or common practices of their hospitals.

Referring to the core components of the WHO IPC programs, this document was set up along three dimensions, addressed separately:

1. Organisational and structural arrangements to implement programs of IPC
2. Targets and methods of HAI surveillance, monitoring and role of feedback, in the context of multimodal prevention strategies
3. Standard Operating Procedures (SOP), methods and effectiveness of healthcare workers educating and training, and interventions on behavioural change and quality of care

Dimensions 2 and 3 were contextualized according to the multimodal prevention strategies. The panel did not take into account the role of antimicrobial stewardship in controlling AMR although recognizing it a fundamental tool and thus referring to other documents [10–13].

## Background

### Epidemiology

HAIs affect millions of patients worldwide every year. The total annual number of patients with an HAI in European acute care hospitals in 2011–2012 was estimated at 3.2 million, causing 37,000 deaths as a direct consequence, more than 2.5 million Disability Adjusted Life Years (DALYs) and 16 million extra days of hospitalization, with an approximate cost of around €7 billion [14].

The 2016–2017 Point Prevalence Survey (PPS) by the European Centre for Disease Prevention and Control (ECDC) has estimated that 8.9 million distinct HAI episodes occur annually in acute care hospitals and long-term care facilities in Europe [15].

In Italy, a number between 450,000 and 700,000 cases of HAI is estimated to occur every year in hospitalized patients, of which 30% considered preventable [16]. The 2016/2017 Italian PPS2 report on HAIs and antibiotic use in acute care hospitals reported that the prevalence of patients with at least one HAI was 8.03%, whereas when considering the average prevalence of HAIs across hospitals, this estimate becomes 6.5%, indicating that some larger, highly specialized hospitals showed a higher prevalence [17–20].

### Hospital-acquired infection sites

The most frequent types of infection include.

1. Catheter-Associated Urinary Tract Infections (CAUTI).
2. Surgical Site Infections (SSI).
3. Hospital-Acquired Pneumonia (HAP) and Ventilator Associated Pneumonia (VAP).
4. Central Line-Associated BloodstreamIinfections (CLABSI).
5. Care-related Skin and Soft Tissue Infections (SSTI).

A brief detail of these is given below:

### Catheter associated urinary tract infections (CAUTI)

Among hospital-acquired urinary tract infections, 70–80% are attributable to the use of an indwelling urinary catheter. Catheter-Associated Urinary Tract Infections (CAUTI) are the most frequently observed conditions, with an incidence 40% of all HAIs [21].

The duration of catheterization is the most important risk factor for developing CAUTI. Therefore, reducing unnecessary catheter placement and minimizing the duration of catheter stay in situ are the main strategies for prevention of CAUTI. Additional risk factors include female sex, older age, diabetes mellitus, renal failure, and malnutrition [22].

Although the proportion of bacteremic individuals who develop symptomatic infection is low, given the high frequency of indwelling urinary catheter use, CAUTI is one of the most common causes of secondary bloodstream infection. CAUTI is the source of approximately 20% of healthcare-acquired bacteraemia episodes in acute care facilities and more than 50% in long-term care facilities [23].

The estimated cost of HAIs in a Polish Intensive Care Unit (ICU) ranges from EUR 10,035 to 22,411. While in the USA, an estimate of 449,334 healthcare-associated CAUTIs per year, is associated with an additional cost of US$749–10,077-9 per admission in 2007 (or an estimated US$3744 when complicated by blood septicaemia) [24].

Comprehensive recommendations have been published to assist acute care hospitals in implementing and prioritizing their CAUTI prevention efforts [25–27].

### Surgical site infections (SSI)

Surgical Site Infections (SSIs) are a major complication during hospitalization, occurring in 2–5% of patients subjected to surgery. These are the second most common type of nosocomial infections caused primarily by *Staphylococcus aureus* resulting in prolonged hospitalization and increased risk of mortality. In most SSIs, the responsible pathogens originate from the patient’s endogenous flora [28].

From the 2017 European Annual Epidemiology Report, the percentage of SSIs ranges from 0.5% to 10.1%, depending on the type of surgical procedure [29].

SSIs are defined as infections occurring up to 30 days after surgery (if no implant is left in place) and affecting either the incision or deep tissue at the operation site. These infections may be superficial involving only skin and subcutaneous tissue of the incision, or deep incisional infections down to the deep soft tissues (fascia and muscle), or infections involving organs and body spaces.

Major patient-related risk factors include advanced age, diabetes mellitus or other chronic diseases, nutritional status, obesity, immunodepression, colonisation with microorganisms (particularly *S. aureus*). Emergency surgery, type of surgery, length and quality of preoperative stay, skin disinfection, inadequate sterilization of surgical instruments and antimicrobial prophylaxis are procedure-related risk factors [30].

Thus, continuous vigilance is required to minimize the incidence of such infections. This requires a systematic approach, with attention to multiple risk factors to reduce the risk of bacterial contamination and improve patient’s defences [31, 32].

The 2017 Centres for Disease Control and Prevention (CDC) guideline provides new and updated evidence-based recommendations for the prevention of SSI and should be incorporated into comprehensive surgical quality improvement programs to improve patient safety [33, 34].

SSIs are responsible for generating significant costs. In 2017, a French cohort showed an average cost of each SSI treatment of approximately €1814; the same year, the Centres for Disease Control and Prevention guidelines estimated the mean cost caused by SSI treatment at $10,443–$25,546 per SSI. This cost depends on many factors including the patient themselves and the type of surgery [35].

### Hospital-acquired pneumonia (HAP) and Ventilator associated pneumonia (VAP)

Hospital-Acquired Pneumonia (HAP) is the second most common infectious complication contracted during hospitalization. The incidence ranges from 5 to 10 cases per 1000 admissions in patients without risk factors, but this estimate may increase can increase 6 to 20-fold in patients admitted to ICU and receiving mechanical ventilation [36].

Ventilator Associated Pneumonia (VAP) is found in 9–27% of mechanically ventilated patients and usually occurs within 48 h after tracheal incubation [37]. The risk of contracting VAP increases proportionally with prolonging duration of both mechanical ventilation and ICU stay. Mortality attributable to VAP ranges from 15 to 50%, with higher mortality rates in surgical patients in the ICU and in patients with average severity scores on admission [38, 39].

The results of a study on the effect of VAP on the prognosis of ICU patients within 90 days and 180 days showed that the 90-day mortality of VAP patients was 33.33% and the 180-day mortality was 37.62%. The 90-day and 180-day mortality rates were higher in the VAP group than in the non-VAP group. The risk of 90-day and 180-day mortalities in VAP patients were 1.465 times (OR = 1.465, 95% CI: 1.188–1.807, *P* < 0.001) and 1.635 times (OR = 1.635, 95% CI: 1.333–2.005, *P* < 0.001) higher than those in non-VAP patients, respectively [40].

New international evidence-based guidelines for the prevention of HAP/VAP have recently been published in Europe and America and provide guidance on the most effective treatments and management strategies for adult patients with HAP and VAP [36, 41].

### Central line-associated bloodstream infections (CLABSI)

The main source of infections in the circulatory stream are due to the implantation of vascular catheters (Central Line-Associated Bloodstream Infection—CLABSI). Catheters are placed in the central line to deliver fluids and medications, but prolonged use can cause severe bloodstream infections resulting in impaired health, and increased hospitalization and cost of care [42]. CLABSIs account for approximately 20% of nosocomial circulatory infections, with a mortality rate of 12–25% [43].

Host factors that increase the risk of CLABSI are chronic disease, immunocompromised states (organ transplantation, diabetes mellitus), malnutrition, total parenteral nutrition, loss of skin integrity (burns), and prolonged hospitalization prior to catheter insertion. Femoral central venous catheters are associated with the highest risk of CLABSI, followed by internal jugular and subclavian catheters. In addition, catheter type, insertion conditions, catheter care, and operator skill also influence the risk of CLABSI.

Of all HAIs, CLABSIs are associated with a high-cost burden of approximately $46,000 per case [44]. Most cases are preventable with appropriate aseptic techniques, surveillance, and management strategies.

The updated guideline released by the CDC in 2011 along with more recent studies highlight new strategies to reduce the incidence of CLABSI [45–47].

### Care-related skin and soft tissue infections (SSTI)

Skin and Soft Tissue Infections (SSTIs) are common in outpatient clinic and emergency department visits and include a wide variety of infections of the various layers of skin, fascia, and muscle. SSTIs usually result from traumatic, surgical, or healthcare-related skin breakdown with secondary infection by microorganisms [48].

The severity of SSTIs ranges from mild and superficial to deeper or potentially fatal necrotizing infections requiring hospitalization or intensive care. Among hospitalized or critically ill patients, several epidemiological studies have shown that about 4.3–10.5% of septic episodes are caused by SSTIs [49, 50].

Using data from the 2000–2004 US Healthcare Cost and Utilization Project National Inpatient Sample, Edelsberg et al*.* suggested that the majority of hospitalized SSTIs were either “superficial” (58.6%) or “deeper and/or healthcare-associated” (40.1%) infections; the percentage of “often fatal” SSTIs was relatively low (1.3%) [51].

In a large database study of skin-related conditions in the ICU, only 0.4% of all ICU admissions had SSTIs, and about 60% of those were necrotizing fasciitis, a potentially fatal infection [52].

Two other studies, including only “superficial” and “deep and/or healthcare-associated” infections, showed that approximately 2.0–5.8% of patients hospitalized with SSTIs are admitted to the ICU [53, 54].

A large number of expert opinions, guidelines, and recommendations for the management of SSTIs have been published over the past decade, taking into account the initial severity of the patient (whether or not the patient requires ICU admission), the extent of the infection (superficial or deep infection), and risk factors for resistant microorganisms essentially related to healthcare-associated circumstances [55–58].

### Factors influencing the development of HAIs

Numerous factors can increase the risk of contracting a HAI, which, in general, can be divided into three groups: host factors, microorganism-related factors, and environmental factors [59].

Patient-related risk factors include advanced age, multiple underlying comorbidities, chronic conditions such as chronic kidney disease, cardio-respiratory disease, and diabetes mellitus, and all conditions of immunosuppression related to drugs (steroid therapy, chemotherapy, radiation therapy, immunomodulatory therapies) and diseases (HIV infection, onco-hematologic diseases, solid organ or bone marrow transplantation, burns, and malnutrition) [60].

Potentially all microorganisms can cause HAI, but generally bacteria and viruses are the main infectious agents. One of the main risk factors related to microorganisms is certainly antibiotic resistance [61].

Finally, the main environmental risk factors are exposure to invasive procedures that increase the risk of infectious complications due to direct access of microorganisms to normally sterile areas of the body and contamination of the devices themselves at the time of use. Exposure to invasive procedures and/or complex surgeries, length of hospital stay, frequent visits to healthcare facilities, mechanical ventilator support, and a stay in an ICU also increase the likelihood of infectious complications [62].

In conclusion, the risk of HAIs depends on the infection control practices in the facility, the condition and immune status of the patient, and the prevalence of various pathogens in the community.

### Mode of transmission

The main route of transmission is by contact. Direct or indirect contact and transmission by droplets fall into this category [63].

Direct contact between a susceptible host and an infected or colonized person can occur during daily care activities, primarily through hands, or through the contact between patients.

Indirect contact occurs through a contaminated intermediate object (instrumentation/surfaces) or through a contaminated common vehicle (food, blood, infusion fluids, disinfectants).

Droplet transmission occurs when droplets containing the pathogens emitted in the act of coughing or sneezing by an infected person are inhaled by a susceptible person who is within a short distance (via droplets) or at a distance (aerosol). Another mode of transmission is by the airborne route, through microorganisms that survive in the air and are transmitted over distance.

## Discussion

The expert panel identified a range of structural, organizational, and management components, given the existing evidence and experts’ opinions, that are crucial to effective implementation of infection control programmes in a hospital setting.

### Core component 1: IPC ORGANIZATION, STRUCTURE

#### Organisational and structural arrangements to implement programs of IPC

Facility’s top management should clearly express the healthcare facility's commitment to address HAIs and antimicrobial resistance (AMR) in a shared and signed document, defining the budget, allocating resources (bed occupancy, staffing, workload, materials and equipment) and how it intends to pursue it in a programme (objectives, activities, timeline, indicators) [62].

As an example, Box 1 shows the annual plan for prevention and control of HAIs of the “Alessandro Manzoni” Hospital in Lecco, Italy.

**Box 1:** Annual plan for prevention and control of HAIs

According to the indications of the Lombardy region, an “Annual Plan for Prevention and Control of Healthcare Associated Infections” has been implemented since 2014 at the “Alessandro Manzoni” Hospital of Lecco with the aim of preventing, monitoring, and controlling healthcare associated infections. The plan is drawn up each year by a dedicated working group and approved by the local Hospital Committee for Healthcare Associated Infections. The working group is managed by the Hospital Medical Director and includes the following operators: infection control nurse, microbiologist, infectious diseases specialist, pharmacist, hygiene specialist, quality and risk managers.
The structure of the annual plan is reported below and includes:
• Actions to promote appropriate hygiene measures (e.g., actions to increase the use of alcohol-based formulations for hand cleaning)
• Guidelines for empirical and targeted therapy based on national recommendations and local epidemiology, especially in life-threatening emergencies (e.g., meningitis and sepsis)
• Actions against the spread of nosocomial infections driven by specific local situations
• Monitoring of alert microorganisms, including methicillin-resistant *Staphylococcus aureus* (MRSA), vancomycin-resistant enterococci (VRE), ESBL-producing *Enterobacterales*, carbapenem-resistant isolates belonging to *Enterobacterales*, *Pseudomonas aeruginosa* and *Acinetobacter baumannii*). The process starts from the microbiology laboratory: when an alert organism is isolated an alert is sent via web to the local IPC team
• Monitoring of antimicrobial use as provided by Hospital Pharmacy with a focus on broad spectrum antimicrobials
• Annual one-point survey to establish the local prevalence of HAIs
• Epidemiological reports on circulating pathogens and antimicrobial resistance; in this regard, specific reports are organized separately for different healthcare areas (e.g., medical, surgical and intensive care settings), as well as for outpatients (including patients from long-term care facilities and from the community)
• Educational annual program pointed to an appropriate antimicrobial use for all the hospital personnel
Of note, following the recommendations of the Lombardy Region, special attention has been directed at monitoring carbapenem-resistant *Enterobacterales* (CRE) and some indicators are verified annually to monitor the trend of this worrying drug resistance, taking into account carbapenemase production (Additional file 1).
Each objective is clearly reported, including results to be achieved, operative times, and specific indicators for evaluating obtained results.
The realization of annual plan objectives is then discussed with healthcare workers during dedicated meetings aimed to present criticisms and improvements.
As a result of activities and actions taken over the years, a more appropriate use of antibiotics has been overall obtained, leading to a lowering in-hospital use of fluoroquinolones and carbapenems. In addition, the good practice of hand hygiene steadily grew, now reaching an overall compliance higher than 70%.

While this strategic plan is the responsibility of the facility's top management, the operational aspects should be handled by a multidisciplinary IPC committee and AMR team.

Indeed, appropriate IPC expertise is required to write procedures or adapt and adopt guidelines at the healthcare facility level [64].

Procedures should be evidence-based and referenced to international or national standards, and adaptation to local conditions should be considered for most effective adoption and implementation.

The IPC program should have:

- clearly defined objectives based on local epidemiology and priorities according to risk assessment and functions to contribute to the prevention of HAI and the spread of AMR in health care;
- dedicated and trained professionals in each acute healthcare facility;
- support from facility management by providing materials as well as organizational and administrative support through the allocation of a protected and dedicated budget;
- good quality microbiological laboratory support.

The key figures who belong to the IPC Committee are the healthcare director, the microbiologist, the infectious diseases specialist, the epidemiologist, the risk manager, the pharmacist, and the director of the health professions; these figures are not available in all healthcare facilities, but they can also be considered and identified as external referents or consultants.

The IPC committee is supported by the operations group (hygienist, microbiologist, infectious disease specialist, ward contact person, infection control nurse) which then implement the given strategies [65, 66].

It is essential to have the commitment of the healthcare facility management to clearly identify these goals, define the activities/actions in the different situations, and monitor that they are carried out [67].

The major objectives and activities of the IPC committee are shown in Table 1 and, as an example the Annual Plan of Healthcare-Associated Infections (Piano Annuale delle Infezioni Correlate all’Assistenza—PAICA) of the Lazio Region is described in Box 2.

**Table 1:** The major objectives and activities of the IPC committee

IPC committee	
Main objectives	Main activities
To provide a strategy to management for the implementation (including unplanned events, such as outbreaks) and improvement of the IPC programme	To meet regularly to discuss the current status of the programme
To ensure monitoring and evaluation of IPC policies	To initiate regular awareness campaigns within the facility
To develop and implement policies, guidelines and procedures relating to IPC and ensuring their currency and accessibility to staff	To keep up to date with the latest evidence and recommendations, with particular regard to Guidelines and Good Practices
To review IPC reports and problems that may cause infections and identify areas for intervention by using surveillance and other data	To communicate clearly with the facility administration on all issues related to infection control
To decide how IPC practices can be applied, based on the amount of available equipment, ensuring that decisions are practical and standard	To adhere to international, national, or regional recommendations and initiatives such as the National Action Plan on Antimicrobial Resistance (PNCAR) [68], the National Prevention Plan (PNP), WHO's “Safe Lives: Clean Your Hands” campaign, European Antibiotic Awareness Day, prevalence surveys, etc
To assess and promote improved IPC practices at all levels of the healthcare facility	
To ensure and monitor appropriate staff training in IPC and safety management	
To ensure implementation of multimodal strategies to achieve IPC practice improvement

**Box 2:** Annual Plan of Healthcare-Associated Infections (Piano Annuale delle Infezioni Correlate all’Assistenza—PAICA) in the Lazio Region [69]

Since 2014, the Health Authority in the Lazio Region stated specific indications targeted to public as well as private hospitals and other healthcare facilities, including long-term care facilities, in issuing a specific annual plan for prevention, monitoring and control of healthcare-associated infections (PAICA). The PAICA is thus the planning tool for the operational activities of the Healthcare-Associated Infections Control Committee (Comitato per il Controllo delle Infezioni Correlate all’Assistenza – CC-ICA) and the Antimicrobial Stewardship Team. Healthcare facilities should program at least five activities pursuant to the strategic objectives indicated by the Regional Health Authority to include those of the National Regional Prevention Plan (PRP) and the National Action Plan on Antimicrobial Resistance (PNCAR):
**Objective A** To spread the culture of Safety of care with specific reference to the Prevention of Infectious Risk
Suggested activities:
• Organize training/information activities for healthcare workers aimed at monitoring and preventing HAIs and good use of antimicrobials
• Develop an Antimicrobial Stewardship model to counter resistance to antibiotics
**Objective B** To improve the appropriateness of care and organization in terms of infectious risk, through the promotion of interventions to improve the quality of services provided and the monitoring and/or containment of HAIs including those carried out by invasive infections by carbapenemase-resistant *Enterobacterales* (CRE)
Suggested activities:
• Perform at least one point prevalence survey of HAIs, i.e., associated with endovascular devices with a focus on Central Venous Catheters (CVCs) and Peripherally Inserted Central Catheters (PICCs)
• Consolidate active surveillance of CRE colonization/infections and other alert MDRO and *Clostridioides difficile*
• Monitor alcohol-based products for hand hygiene and anti-infective drug consumption (expressed as Liter or DDD/ hospitalization days, respectively)
• Implement measures to control nosocomial transmission of MDRO colonization/infection
• The annual PAICA should be published on the own facility web site, and should report the results coming from the activities performed in the previous year

### Core component 2: IPC SURVEILLANCE, FEEDBACK

#### Targets and methods of HAI surveillance, monitoring and role of feedback

Surveillance of HAI is critical to inform and guide IPC strategies. Healthcare facility surveillance should be based on national recommendations and standard definitions and customized to the facility according to available resources with clear objectives and strategies.

Active and passive surveillance programs should be implemented to assess and monitor the extent and trends of HAIs, inform alert and precautionary programs, and improve performance, strategy, and skill development [70, 71].

Surveillance should provide information for:

- Description of the status of HAIs (i.e., incidence and/or prevalence surveys, type, aetiology and, ideally, data on severity and attributable burden of disease)
- Identification of the most relevant AMR susceptibility patterns
- Identification of high-risk populations, procedures and exposures
- Early detection of clusters and outbreaks (contact tracing)
- Evaluation of the impact of interventions.

To detect infections, active surveillance includes targeted screening to identify colonized patients on hospital admission, such as performing entry rectal swabs for prevention and control of CRE infections and nasal swabs for prevention and control of MRSA. Active surveillance may be more directly associated with monitoring and controlling the risk of drug-resistant pathogen outbreaks [72, 73]. Monitoring should include all gram-negative and gram-positive organisms (e.g., MRSA) that represent a relevant threat according to local or national epidemiological assessment. In Italy, indicators for MDRO monitoring are particularly focused on CRE, according to the recommendations of the Italian Ministry of Health and European Health Authorities, due to the current worsening of the epidemiological situation in the European Union [74].

Box 3 reports the monitoring and control program of the University Hospital “Luigi Vanvitelli” in Naples for the management of MDR-carriers.

**Box 3:** Monitoring and control program for the management of MDR-carriers

At the “Luigi Vanvitelli” teaching Hospital in Naples, a persuasive-educational program on the management of patients MDR-carriers was initiated in 2019. It was based on audit and feedback conducted by a team of consultants in infectious diseases, clinical microbiology, and hygiene. The departments included in this program were those managing high-risk patients, specifically, nephrology, oncology, geriatrics, infectious diseases, general surgery, and 2 ICUs. All the consultants were responsible for writing and sharing protocols for the screening and the management of the MDR-carriers: all patients in the wards participating in the program were screened for the rectal presence of CRE and nasal presence of MRSA on admission and every 7 days during the hospitalization. The microbiology unit informed infectious disease and hygiene specialists of a positivity to allow for better management of the patient; these specialists performed audits within 48 h to evaluate the management of isolation according to approved protocols, and adherence to these indications was assessed. This program, through collaborative work among consultants in infectious diseases, clinical microbiology, and hygiene, has improved the awareness of staff in the departments included in the program about the management of MDR carriers.	

The responsibility for planning and conducting surveillance and analyzing, interpreting and disseminating the collected data remains usually with the IPC committee.

Passive surveillance consists of data that are routinely generated from patient registration, laboratory or pharmacy data, or data from discharge. Quality microbiology and laboratory capacity are essential to enable reliable HAI surveillance, and laboratory reporting of alert organisms, usually multi-resistant, is a due act of surveillance within the facility [75].

A laboratory-based surveillance protocol for the detection and management of MDRO clusters is reposted in Box 4.

**Box 4:** Surveying and Managing MDRO cluster

A multidisciplinary IPC team has been established at the National Institute for Infectious Diseases “Lazzaro Spallanzani” in Rome in order to implement measures to prevent and control HAIs. A specific protocol concerns the laboratory-based surveillance of the following MDR alert organisms (carbapenem-resistant *Acinetobacter spp.*, *Klebsiella spp.*, *Escherichia coli* and *Pseudomonas aeruginosa*; MRSA; VRE) and *Clostridioides difficile*. After receiving an alert, the operative group of the IPC team performs a targeted epidemiological investigation, the results of which are discussed by the IPC team in order to implement corrective measures. In accordance with the hospital’s surveillance programme aimed at detecting carbapenem-resistant bacteria, rectal swabs were taken in the ICU from all patients on admission and then once weekly for the entire duration of their stay.
From December 2016 to April 2017, 13 alerts referring to carbapenem-resistant *Acinetobacter baumannii* (CRAB) isolates were collected from seven ICU patients. Epidemiological data, including time of infection and transfer to other wards, patient movements within the ICU and occupied rooms, strongly suggest a potential cluster. CRAB is a well-known nosocomial pathogen causing serious, often life-threatening, infections and outbreaks. To confirm the cluster hypothesis, the IPC team decided to perform molecular typing on the 13 isolates: 9 cultured from clinically relevant samples and the remaining 4 obtained from rectal swabs. Whole Genome Sequencing (WGS) and traditional multilocus typing (MLST) revealed the presence of two types of clusters. These results allowed to identify two patients who were likely the source of two separate transmission chains. The two patients came from two distinct hospitals and shared a narrow temporal and spatial overlap of their stay, supporting the conclusion that two separate transmission events are likely to have occurred.
Immediately following the alarm, investigations were undertaken to reveal possible breaches in the isolation precautions employed, but nothing was found. However, to contain the outbreak, isolation precautions, room and equipment cleaning, and disinfection procedures were audited and reinforced through an on-the-job education session. No other cases occurred either in the ICU or in the clinical wards where patients were subsequently transferred.

The role of pharmacy and the Antimicrobial Stewardship team is to track trends in antibiotic consumption (DDD/100 hospital days) also measured for economic reporting purposes and in hydroalcoholic solution consumption (L/100 hospital days) as a direct indicator for verification of implementation of multimodal strategies related to the use of alcohol-based products [76].

### Core component 3: IPC EDUCATION, TRAINING

#### Standard operating procedures, methods and effectiveness of healthcare workers educating and training, and interventions on behavioural change and quality of care

IPC education and training should be part of an overall healthcare facility educational strategy, including orientation of new employees and provision of continuous educational opportunities for existing staff, regardless of level or position, and assessment and monitoring of good clinical care practices through audits. Education has a positive impact on retention of knowledge, attitudes and practices in all the categories of staff and educational approaches should be informed by behavioural change theories and methods. Teaching the basic concepts and theories of microbiology, infectious diseases and IPC, using a range of educational modalities to maximize the impact of practical and in-service training [77, 78].

Education and training programmes should be audited against predefined checklists that are revised over time to take into account local barriers and behaviour. Education and training should be combined with knowledge tests, competency assessments, or both.

All healthcare workers must be familiar with and fully aware of standard operating procedures (SOPs) for potentially infectious patient isolation and modes of transmission and must verify and monitor their effective implementation. In order to reduce the incidence of nosocomial infections, compliance with interventions is mandatory [79, 80].

Documents may include those presented in the form of specific SOPs such as hand hygiene, environmental hygiene and instruments (decontamination, sterilization), laundry hygiene and waste, water and air hygiene (e.g., Legionnaires' disease), asepsis techniques for invasive procedures and surgery, or in the forms of BUNDLES such as vascular access management and respiratory care management. Moreover, it is important to know the protocols for prevention and protection of personnel, and the principles of good use of antibiotics [12].

Hand hygiene initiatives of the Fondazione Policlinico Universitario “Agostino Gemelli” IRCCS in Rome are described in Box 5.

**Box 5:** Hand hygiene initiatives for IPC

**Fig. 1:**
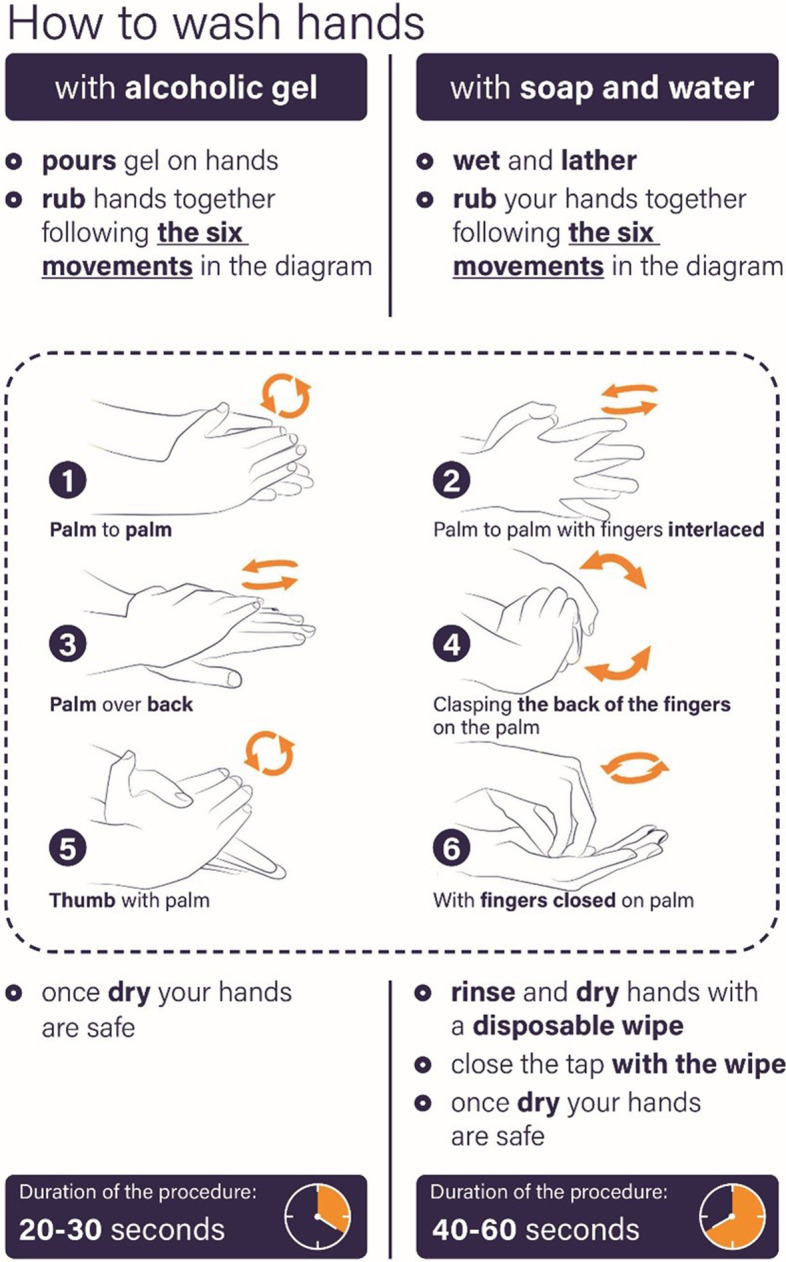
The six steps for a good hand washing (*Courtesy of G. Sganga—Fondazione Policlinico Universitario A. Gemelli IRCCS, Rome*)

**Fig. 2:**
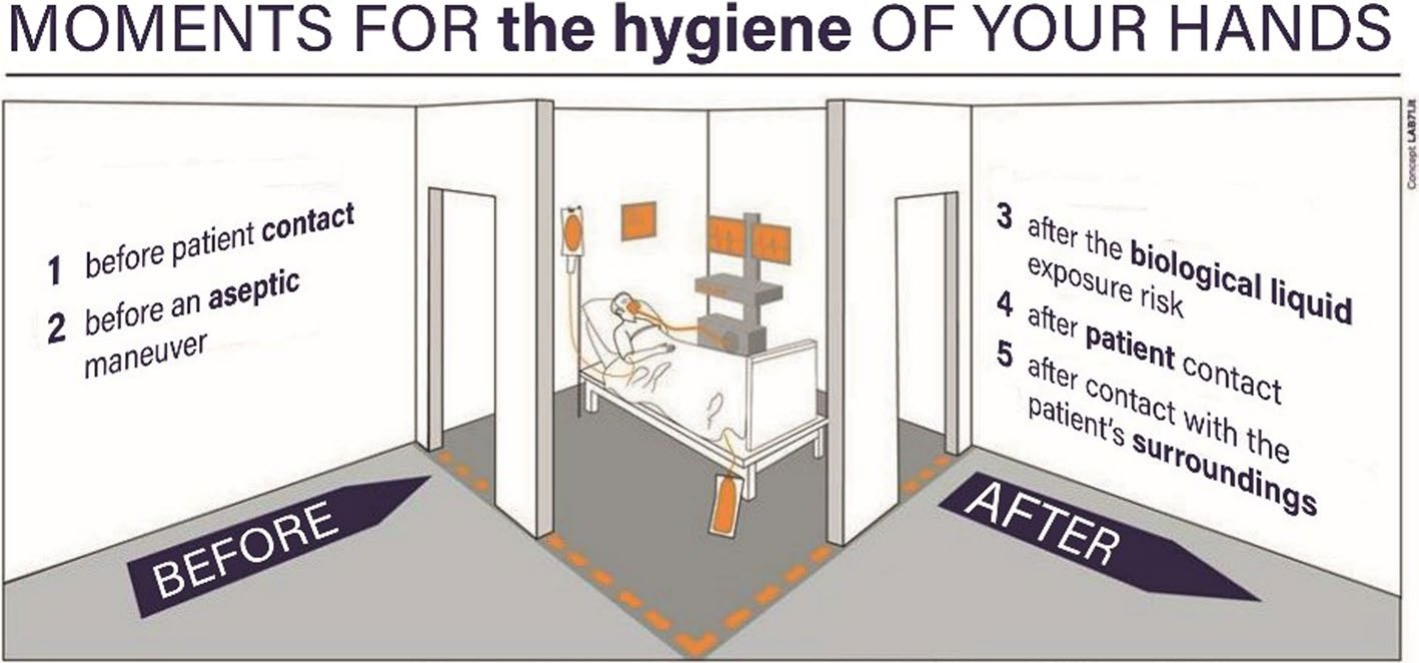
Infographic that summarizes the moments for the hand hygiene inside or outside the patient's room (*Courtesy of G. Sganga—Fondazione Policlinico Universitario A. Gemelli IRCCS, Rome*)

Fondazione Policlinico Universitario “Agostino Gemelli” IRCCS in Rome has always been very attentive to Infection Prevention and Control (IPC) and since 2014 has been collaborating with the Catholic University’s Master’s program “Sepsis in Surgery” promoting an intensive campaign on handwashing for IPC.
The hospital is participating in World Hand Hygiene Day launched by WHO, which is held annually on May 5. During this day, the hospital's entire infection control team (microbiologists, infectious disease and hygiene specialists, intensivists, surgeons and many other professionals) enthusiastically participates in different events organized within the campus, from lectures to demonstrative activities involving physicians and nurses [81, 82].
Moreover, several initiatives have been implemented, including:
• the draft of a series of posters promoting the good practice of hand hygiene to prevent transmission of pathogens
• exemplification and validation of the practice of handwashing by reducing the number of steps (only six) required for good cleaning as shown in the Fig. 1. These steps were actively promoted in a variety of media including posters, gadgets (on 6 faces of a dice), pamphlets, videos
• the creation of an infographic that summarizes when hands should be washed according to the activities performed inside or outside the patient's room as shown in the Fig. 2
As a result of these activities and actions taken by the hospital managers, the good practice of hand hygiene is growing steadily, now reaching an overall compliance of more than 80%
In addition, all of these communication, education and training activities, along with the regular surveillance of infections in the various departments by the hospital's Infection Control Committee, help to increase attention to and awareness of infection risk in the hospital and raise the level of the quality process.
Once again, this dissemination of knowledge and the corresponding adherence to correct clinical practices in terms of prevention and control of hospital infections has proven to be of particular importance during this long period of Covid-19 pandemic.

## Conclusion

Implementation of the WHO recommendations on the "core components" of IPC is necessary to build functioning programs that lead to the effective reduction of HAIs.

Referring to the core components of the WHO IPC programs, this expert opinion statement was set up along three dimensions, addressed separately: (1) organizational and structural arrangements to implement IPC programs with clearly defined objectives, functions, and activities for the purpose of preventing HAIs through IPC good practices; (2) targets and methods of HAI surveillance, monitoring, outbreak management, and role of feedback; (3) methods and effectiveness of healthcare workers educating and training.

As a result of the activities and actions taken by some Italian hospitals described in the boxes, for example, a more appropriate use of antibiotics has been achieved, leading to a decrease in in-hospital use of fluoroquinolones and carbapenems; good practice of hand hygiene has increased, reaching an overall compliance of more than 80%; and a specific protocol on laboratory-based surveillance of MDR alert organisms has been developed, leading to better management of MDR carriers.

In conclusion, sharing independent suggestions on an effective risk management plan and comparing clinical experiences can be helpful in identifying interventions and actions needed to monitor and contain HAIs.

It is important to clarify that this document is based only on Italian experiences and practices in HAI prevention and control and cannot represent universal recommendations. However, the interventions and strategies proposed here to improve the quality and safety of care with respect to infectious risk can be applied to other countries, including those with low resources.

## Supplementary Information

**Additional file 1.** Indicators directed at monitoring Carbapenemase-producing *Enterobacterales* (CPE) and Carbapenem-resistant *Enterobacterales* (CRE).

## Data Availability

Not applicable.

## Abbreviations

AMR: Antimicrobial resistance
CAUTI: Catheter-Associated Urinary Tract Infection
CDC: Centres for Disease Control and Prevention
CLABSI: Central Line-Associated Bloodstream Infection
CPE: Carbapenemase-producing *Enterobacterales*
CRAB: Carbapenem-resistant *Acinetobacter baumannii*
CRE: Carbapenem-resistant *Enterobacterales*
ECDC: European Centre for Disease Prevention and Control
HAI: Healthcare-Associated Infection
HAP: Hospital-Acquired Pneumonia
ICU: Intensive Care Unit
IPC: Infection Prevention and Control
PAICA: Piano Annuale delle Infezioni Correlate all’Assistenza (Annual Plan of Healthcare-Associated Infections)
PNCAR: National Plan to Combat Antimicrobial Resistance
PPS: Point Prevalence Survey
SOP: Standard Operating Procedures
SSI: Surgical Site Infection
SSTI: Skin and Soft Tissue Infection
VAP: Ventilator Associated Pneumonia
WGS: Whole Genome Sequencing
WHO: World Health Organization
