# Supplementary material for: Pillars for prevention and control of healthcare-associated infections: an Italian expert opinion statement
Source: Antimicrob Resist Infect Control. 2022 Jun 20;11:87. doi: 10.1186/s13756-022-01125-8 (PMC9207866; doi:10.1186/s13756-022-01125-8)
Supplement: Supplementary file 1 — Additional file 1. Indicators directed at monitoring Carbapenemase-producing Enterobacterales (CPE) and Carbapenem-resistant Enterobacterales (CRE). [file 13756_2022_1125_MOESM1_ESM.docx]

**Supplementary material**

**Indicators directed at monitoring carbapenemase-producing Enterobacterales (CPE) and carbapenem-resistant Enterobacterales (CRE).**

• Number of CPE isolates from clinical samples/1000 admissions

• Number of CPE isolates from clinical samples /10 000 hospitalization days

• Number of CPE isolates from rectal swabs/1000 screened patients

• Number of CPE isolates from rectal swabs/10 000 hospitalization days

• Number of CPE isolates from blood cultures/1000 admissions

• Number of CPE isolates from blood cultures/10 000 hospitalization days

• Number of CPE isolates from urine samples/1000 admissions

• Number of CPE isolates from urine samples/10 000 hospitalization days

• Percentage of carbapenem-resistant Enterobacterales/total isolates of Enterobacterales

• Percentage of carbapenem-resistant Klebsiella pneumoniae/total isolates of K. pneumoniae
